# Comparison of Culture Results between Specimens from Corneal Scraping with Microhomogenization and Corneal Swab in Moderate and Severe Bacterial Corneal Ulcers

**DOI:** 10.1128/spectrum.03565-22

**Published:** 2023-03-21

**Authors:** Faraby Martha, Lukman Edwar, Anis Karuniawati, Ahmad Fuady, Ramadhiana Maktazula Tuasikal

**Affiliations:** a Department of Ophthalmology, Faculty of Medicine, Universitas Indonesia, Cipto Mangunkusumo Hospital, Jakarta, Indonesia; b Department of Clinical Microbiology, Faculty of Medicine, Universitas Indonesia, Cipto Mangunkusumo Hospital, Jakarta, Indonesia; c Department of Community Medicine, Faculty of Medicine, Universitas Indonesia, Jakarta, Indonesia; Institut National de Santé Publique du Québec

**Keywords:** bacterial cultures, scraping, microhomogenization, corneal swab

## Abstract

The purpose of this study was to compare the bacterial culture results from corneal infiltrate specimens collected using two different methods, corneal scraping followed by microhomogenization and corneal swab. This is a comparative crosssectional study, with 18 participants undergoing corneal specimen collection using each of the 2 sampling techniques. The results from the scraping and swab methods were separated and then compared using data analysis. The proportion of Gram stain results that matched the culture results for the scraping-microhomogenization technique was 6/13 (46.2%), and the proportion of Gram stain results that matched the culture results for the swab technique was 5/13 (38.5%) (McNemar test *P* value, 1.000; *P* > 0.05). The proportion of positive cultures obtained using the scraping-microhomogenization technique was 13/18 (72.2%), and the proportion of positive cultures obtained using the swab technique was 9/18 (50%) (McNemar test *P* value, 0.219; *P* > 0.05). The Kappa suitability test value for comparison of the scraping-microhomogenization technique to the corneal swab was 0.333. The specimens collected by corneal scraping followed by microhomogenization had a higher positive bacterial culture rate than those collected by corneal swab, but the results were not statistically significant.

**IMPORTANCE** This study aimed to compare the culture results between scraping specimens with microhomogenization and corneal smears in patients with moderate to severe bacterial corneal ulcers. This study could be a guideline for treating bacterial corneal ulcers.

## INTRODUCTION

Bacterial corneal ulcer is a defect in the corneal epithelium with loss of stroma tissue and/or inflammation of the stroma caused by bacteria (1, 2). Bacterial infection is the most common cause of corneal ulcers and can cause visual impairment at various ages. The number is expanding with the increasing use of contact lenses, topical steroids, trauma, and abnormalities of the eye surface that cause blindness due to corneal scar, perforation, or endophthalmitis (3, 4).

Examination of bacterial culture as the gold standard treatment for bacterial corneal ulcers is not 100% able to grow germs. Its success ranges from 37% using a sterile 23 gauge needle to 84% with a flame-sterilized platinum spatula or a calcium alginate swab (5, 6). In our hospital, the positive culture rate from 220 presumed bacterial corneal ulcer patients was 41.8%. A common method used in the removal of the corneal specimen is a corneal biopsy using a 26 gauge needle in combination with a corneal (7).

There are two commonly used techniques for collecting corneal ulcer specimens, corneal swab and corneal biopsy or scraping (8). The corneal swab is more practical and effective for collecting specimens and provides better bacterial absorption and release onto the solid agar medium than a spatula does (8). Using corneal scraping, tissue is collected from the corneal edge and the ulcer base, so it is expected that a sufficient amount of bacterial sample is collected and access to the infection in deeper corneal stroma is obtained, rather than just swabbing the corneal surface (9). Corneal scraping can be combined with the microhomogenization technique. Using a scraping technique followed by microhomogenization provides 20% more positive cultures than using the usual technique of corneal scraping (10).

Knowing the microorganism that causes a corneal infection is very important to the success of therapy. This study aimed to compare the culture results from scraping biopsy specimens with microhomogenization and the results from corneal smears in patients with moderate to severe bacterial corneal ulcers. This study could be a guideline in treating bacterial corneal ulcers.

## RESULTS

A total of 18 patients were included in this study, divided into two groups of 9 patients each. The number of male subjects was more dominant than those of female subjects but no significant difference between the two groups (Table 1).

**TABLE 1:** Participant characteristics^*a*^

Variable	No. (%) of participants in:			*P* value
	Group A	Group B	Total	
Age (yrs)				0.375^*b*^
Median (range)	27 (18–53)	47 (18–62)		
Gender				
Male	5 (55.6)	7 (77.8)	12 (66.7)	0.620^*c*^
Female	4 (44.4)	2 (22.2)	6 (33.3)	
Laterality				
Right	4 (44.4)	5 (55.6)	9 (50)	1.000^*c*^
Left	5 (55.6)	5 (55.6)	9 (50)	
History of antibiotic use				
Yes	7 (77.8)	7 (77.8)	14 (77.8)	1.000^*c*^
No	2 (22.2)	2 (22.2)	4 (22.2)	
Ulcer grade				
Moderate	4 (44.4)	2 (22.2)	6 (33.3)	0.620^*c*^
Severe	5 (55.6)	7 (77.8)	12 (66.7)	

a Total, *n* = 18 participants; group A (scraping followed by swab), *n* = 9 participants; group B (swab followed by scraping), *n* = 9 participants.

b Mann-Whitney test.

c Fisher’s exact test.

The percentage of positive cultures aims to assess the effectiveness of both examination techniques. Table 2 shows that that the positive culture results of the scraping-microhomogenization technique were higher than those of the swab technique (72.2% versus 50%, respectively); for participant B8, 2 bacteria grew from the scraping specimen, while none grew from the swab specimen. There were 4 culture results with the same bacteria between the two groups, and two of them were Pseudomonas aeruginosa.

**TABLE 2:** Positive culture using the scraping-microhomogenization and swab techniques

Participant no.	Microorganism(s) obtained using:	
	Scraping-microhomogenization	Swab
A1	Pseudomonas aeruginosa	Pseudomonas aeruginosa
A2	Micrococcus luteus	None
A3	Micrococcus luteus	Staphylococcus epidermidis
A4	None	None
A5	Stenotrophomonas maltophilia	None
A6	Serratia marcescens	Serratia marcescens
A7	Streptococcus pneumoniae	Streptococcus pneumoniae
A8	Staphylococcus capitis	Staphylococcus epidermidis
A9	None	None
B1	Pseudomonas aeruginosa	Pseudomonas aeruginosa
B2	Micrococcus luteus	None
B3	None	None
B4	Staphylococcus xylosus	None
B5	None	None
B6	Micrococcus luteus	Propionibacterium acne
B7	Micrococcus luteus	Staphylococcus epidermidis
B8	Ochrobactrum anthropi, Staphylococcus epidermidis	None
B9	None	Staphylococcus epidermidis
Total no. (%) of positive cultures	13/18 (77.2)	9/18 (50.0)

As Table 3 shows, the proportion of Gram stain and culture results that matched for the scraping-microhomogenization technique was 6/13 (46.2%), and the proportion of Gram stain and culture results that matched for the swab technique was 5/13 (38.5%). The McNemar test *P* value was 1.000 (*P* > 0.05); statistically, there was no difference in the proportions of Gram stain and culture results that matched for the scraping-microhomogenization and swab techniques. The kappa suitability test value for comparison of the scraping-microhomogenization technique to the swab technique was 0.217 (fair agreement, or “sufficient”). This value was lower than the expected suitability, the “good” level of compliance (kappa value, 0.61 to 0.80). The kappa *P* value was 0.429 (>0.05), or not statistically significant.

**TABLE 3:** Comparison of the proportion of microscopic examination (Gram stain) and culture results that matched for the scraping and swab specimens

Swab results	No. (%) of scraping specimens			*P* value^*a*^
	Matching culture results	Not matching culture results	Total	
Matching culture results	3 (23.1)	2 (15.4)	5 (38.5)	1.000
Not matching culture results	3 (23.1)	5 (38.5)	8 (61.5)	
Total	6 (46.2)	7 (53.8)	13 (100)	

a McNemar test.

Table 4 shows the relationship between the collection techniques and positive culture results. The proportion of positive cultures using the scraping-microhomogenization technique was 13/18 (72.2%), while the proportion of positive cultures using the swab technique was 9/18 (50%) (McNemar test *P* value, 0.219; *P* > 0.05). The kappa suitability test value for comparison of the scraping-microhomogenization technique to the swab technique was 0.333 (fair agreement, or sufficient). This value was lower than the expected suitability, the good level of compliance (kappa value, 0.61 to 0.80). The kappa *P* value was 0.114 (>0.05), or not statistically significant.

**TABLE 4:** Comparison of positive cultures from scraping-microhomogenization and swab specimens

Swab culture result	No. (%) of scraping-microhomogenization cultures			*P* value^*a*^	Kappa value	*P* value^*b*^
	Positive	Negative	Total			
Positive	8 (44.4)	1 (5.6)	9 (50)	0.219	0.333	0.114
Negative	5 (27.8)	4 (22.2)	9 (50)			
Total	13 (72.2)	5 (27.8)	18 (100)			

a McNemar test.

b Kappa test.

## DISCUSSION

A comparative cross-sectional audit of 18 corneal scrapes undertaken at Cipto Mangunkusumo Hospital between July and November 2016 demonstrated that the proportion of Gram stain-positive samples collected by corneal scraping was 6/13 (46.2%), while the proportion of Gram stain-positive samples collected by swabbing was 5/13 (38.5%), which is not statistically significant (*P* > 0.05). The correspondence between the two techniques in terms of Gram according to culture has a Kappa suitability test value of 0.217 (fair agreement or “sufficient”) with a value of *P* > 0.05. This result may have been caused by the differing locations of material collection for the two techniques. Using the scraping technique, the corneal specimen is collected from beneath the surface and debris from the edge and base of the ulcer, while using the swab technique, the corneal specimen is collected from the surface of the ulcer. Data on the suitability of Gram staining for cultures with low bacterial concentrations were also obtained from previous descriptive studies in our hospital using corneal swabs, which stated that 23.5% of results indicating Gram-negative rods and 66.7% of results indicating Gram-positive cocci were not in accordance with the results of their culture (7). Another study also reported that the results of the Gram examination were incompatible with the culture results for bacterial corneal ulcers; only 57% of the Gram staining results were compatible with the culture (11). This may be caused by several factors, such as too little biopsy material and the possibility of errors during specimen collection, transport of the medium to the laboratory, or when the medium is examined in the laboratory. For patients who have previously been given empirical antibiotic therapy, Gram examination and culture also yield negative results (1, 11).

The total number of positive cultures in this study was 14/18 (77.8%), which was slightly different when each technique was assessed separately (72.2% using the scraping-microhomogenization technique, 50% using the swab technique). The positive culture results for the scraping-microhomogenization technique in this study were similar to those achieved using the same technique in a study by Diamond et al., 71% (10). Additional microhomogenization procedures theoretically can cover up the weakness of the corneal scraping technique, that is, the small sample volume and uneven sample distribution on the agar culture medium (10).

If the first culture result is negative in a patient with suspected bacterial infection, a repeat culture may be performed using scraping-microhomogenization, as in 33% of samples in group B. This is considered to be clinically significant and shows the superiority of the latter examination technique (10).

The most common isolates obtained in positive culture samples in this study were Gram-positive cocci (14/22; 63%); this finding is in agreement with studies in India (69.4%) and Africa (68.9%). The next most common isolates in this study were Gram-negative rods (7/22; 32%), results similar to those of studies in India (30.6%) and Africa (17%) (12). The most common bacterial species found in this study were Staphylococcus epidermidis (22.7%), Micrococcus luteus (22.7%), and Pseudomonas aeruginosa (18%). In a study in India, the most common bacterial species belonged to the genera Streptococcus (46.8%), Staphylococcus (24.7%), and Pseudomonas (14.9%). A study in Africa found that the most common bacterial species belonged to the genera Pseudomonas (52.5%), Streptococcus (20%), and *Corynebacterium* (10%) (13).

In several studies, Staphylococcus spp. were the most common bacteria in bacterial corneal ulcers. In this study, Staphylococcus epidermidis was the most common bacterium (22.7%). This is similar to the findings of the study in Africa, where Staphylococcus epidermidis was the most common bacterium found in bacterial corneal ulcers, at 27% (12).

In one patient with risk factors for diabetes mellitus, Ochrobactrum anthropi bacteria were found in culture isolates using microhomogenization scrapings. Ochrobactrum anthropi belongs to a group of Gram-negative rods that have been reported as a cause of corneal ulcers and are aggressive in immunocompromised patients (14). This bacterium has also been reported as a cause of endophthalmitis.

There were no complications such as corneal perforation during the sampling in this study. This is in agreement with the studies of Diamond et al. and Krishna et al. (10, 12), which showed that the safety level of the corneal scraping technique was quite good.

### Conclusion.

Bacterial cultures of specimens collected by corneal scraping followed by microhomogenization had a higher positive culture rate (72.2%) compared to that of bacterial cultures of specimens collected with a corneal swab (50%), but these results were not statistically significant (*P* > 0.05), with a kappa value of 0.333.

The application of corneal scraping followed by microhomogenization for specimen collection in patients with moderate and severe bacterial corneal ulcers has clinical advantages, although they are not statistically significant (15, 16).

## MATERIALS AND METHODS

This was a comparative cross-sectional study, with 18 participants. Each participant underwent corneal specimen collection using 2 sampling techniques, both corneal scraping with microhomogenization and a corneal swab, to enable paired analysis of the culture results and matching of the Gram smear with the corneal culture. The samples were collected consecutively, with the order in which they were collected switched between the groups: group A underwent scraping-microhomogenization and then a swab, and group B underwent a swab and then scraping-microhomogenization.

The inclusion criteria were bacterial corneal ulcer (diagnosis based on clinical course) classified as moderate or severe according to the modified Jones classification (17); age over 18 years old; willingness to participate in the study; and direct smear examination of Gram stain and KOH test results with a finding of either bacterial cells without fungal hyphae or no bacterial cells or fungal hyphae. The exclusion criteria were clinical perforation or impending perforated bacterial corneal ulcer; endophthalmitis; corneal ulcer with clinical and Gram stain support for a bacterial cause but with a positive fungal culture as well; and samples that could not be examined in the laboratory for any reason. All patients involved in this study provided informed consent.

The patients were given 0.5% pantocain eye drops for corneal anesthesia. A corneal swab was performed for a direct smear for Gram staining and a KOH test. Corneal scrapings were collected from the edge and base of the ulcer under aseptic conditions using the tip of a 1-cc tuberculin syringe bent at 60 degrees under slit lamp magnification and inserted into a sterile container. The corneal swab was collected with a sterile cotton swab and inserted into the Stuart transport medium.

For patients in group A, corneal scraping was followed by microhomogenization of the specimen until a volume of 80 μL was obtained; the solution was inoculated onto the culture medium. A corneal swab specimen was then collected from the same eye and directly inoculated onto the culture medium. In contrast, the patients included in group B had these samples collected in reverse sequence. Specimens from the 2 collection techniques were inoculated onto the surface of the solid medium. The culture medium was incubated at 35°C for 20 h. The isolates grown were identified biochemically and subjected to antibiotic susceptibility testing using the Vitek2 automated machine.
